# Assessing Student Satisfaction With Teaching-Learning Methods in Postgraduate Anaesthesiology: Nationwide Survey Based Analysis

**DOI:** 10.7759/cureus.116697

**Published:** 2026-09-21

**Authors:** Anshul Jain, Manish K Singh, Apurva A Mittal, Reena K, Gaurav Jain, Renu Sinha, Shubham Achari, Brajendra Verma

**Affiliations:** 1 Department of Anaesthesiology, Maharani Laxmi Bai Medical College, Jhansi, IND; 2 Department of Anaesthesiology and Critical Care, King George's Medical College, Lucknow, IND; 3 Department of Anaesthesiology, Sarojini Naidu Medical College, Agra, IND; 4 Department of Anaesthesiology, Institute of Medical Sciences, Banaras Hindu University, Varanasi, IND; 5 Anaesthesiology and Critical Care, All India Institute of Medical Sciences Rishikesh, Rishikesh, IND; 6 Department of Anaesthesiology, All India Institute of Medical Sciences, New Delhi, IND

**Keywords:** anaesthesiology education, learning resources, postgraduate medical training, student perception, teaching methodologies

## Abstract

Background

Teaching methodologies in postgraduate anaesthesiology education are diverse, with equal weightage given to conventional methods such as lectures, seminars, tutorials, case presentations, and demonstrations. This nationwide survey aimed to compare student perceptions of these teaching methods and identify students' preferences to enhance the educational experience.

Methods

A cross-sectional survey was conducted from November 16, 2023, to August 10, 2024, among postgraduate anaesthesiology students and senior residents in India. A structured questionnaire was distributed via Google Forms, utilizing a five-point Likert scale to evaluate teaching methods. Statistical analyses, including analysis of variance (ANOVA) and Tukey’s Honestly Significant Difference (HSD) tests, were used to determine differences in satisfaction levels across methods.

Results

A total of 1,174 eligible participants were included in the analysis. Tutorials and Demonstrations (19.4% rated “very interesting”) and Case Presentations (18.8% rated “very interesting”) were perceived as the most engaging teaching methods, whereas Seminars (3.9%) and Journal Clubs (5.5%) were perceived as the least engaging. Student satisfaction with class frequency was highest (85.2%) for schedules comprising three sessions per week.

Conclusions

Postgraduate anaesthesiology students perceived teaching methods differently, with interactive teaching methods being rated as more interesting than didactic methods. Incorporating postgraduate student feedback into teaching programmes may enhance learner engagement and improve postgraduate teaching.

## Introduction

Anaesthesiology is a multidisciplinary specialty encompassing perioperative care, emergency medicine, intensive care, and pain management, requiring postgraduate training across diverse clinical settings. In India, anaesthesiology has one of the highest postgraduate intakes, ranking second only to General Medicine in the number of postgraduate seats [1]. The breadth and complexity of this training underscore the importance of effective teaching-learning methods in postgraduate anaesthesiology education.

The current landscape of postgraduate anaesthesiology education in India, as governed by the guidelines of the National Medical Commission, includes conventional teaching methods such as lectures, seminars, tutorials, demonstrations, and case presentations, with comparable weightage assigned to each [2,3]. Internationally, postgraduate medical education has increasingly incorporated learner-centred and interactive approaches, including case-based, problem-based, and simulation-based learning, to promote active participation and application of knowledge to clinical practice [4,5]. However, such contemporary approaches have not been uniformly integrated into postgraduate anaesthesiology curricula. The Objective Structured Clinical Examination (OSCE), through its formative use with structured feedback, has also emerged as a teaching-learning tool and is being used in some institutions [6-8].

Given the diverse learning preferences of postgraduate students, we hypothesized that their perceptions of teaching methods would vary in terms of interest, engagement, and perceived effectiveness. Understanding these differences can inform strategies to improve postgraduate anaesthesiology teaching.

Aligned with this hypothesis, the primary aim of this study was to identify the most and least interesting teaching methods as perceived by postgraduate anaesthesiology students. The secondary objectives were to identify the factors influencing student engagement and satisfaction, gather student feedback on current teaching practices and areas for improvement, and assess students’ perceptions of the educational value of different teaching methods. The study also explored the potential application of artificial intelligence (AI) in generating an illustrative teaching schedule based on aggregated student preferences.

To accomplish these objectives, the authors conducted a nationwide survey targeting a comprehensive cohort of postgraduate anaesthesiology students. In recent years, the use of Google Forms for survey administration has gained recognition as an effective tool in educational research [9,10]. The platform facilitates accessible, efficient, and structured questionnaire distribution and data collection.

## Materials and methods

Study design and participants

The study was conducted as a national internet survey. The Institutional Ethics Committee of Maharani Laxmi Bai Medical College (Certificate No. 9616) approved the study on September 26, 2023. The protocol adhered to ethical standards and the Declaration of Helsinki. The target group included students pursuing postgraduate training in anaesthesiology in India and those who had completed their postgraduate qualification within the preceding three years and were currently working as senior residents in a medical college. Responses from the participants who did not meet these eligibility criteria, as well as incomplete responses, were excluded from the analysis. Informed consent was obtained through completion of the survey, as stated in the survey instructions: “Filling the form would be considered as positive consent for the same.”

Constructing the survey

The study utilized a structured questionnaire administered through Google Forms (the complete survey is presented in the Appendices). The questionnaire was developed based on literature review, previous survey methodologies, investigator experience, and peer discussions [11,12].

The questionnaire underwent content validation through multiple rounds of expert review by Professors of Anaesthesiology who had successfully completed the Basic Course in Medical Education Technology (BCMET) accredited by the National Medical Commission (NMC). A pilot test involving volunteer postgraduate students was conducted to assess the clarity and comprehensibility of the questionnaire before finalization. The complete questionnaire is provided in the Supplementary File.

The questionnaire began with basic demographic questions, including the participant's state or union territory, followed by a well-validated five-point bidirectional Likert scale that allowed participants to rate each teaching method from “Very Boring” to “Very Interesting” [13,14] (Table 1)​​. To ensure that ratings were based on direct exposure, participants were first asked to confirm whether each teaching method (e.g., Seminars, Lectures, Tutorials, Demonstrations, Case Presentations) was part of their actual teaching schedule. Students could rate a method only if they confirmed its inclusion in their curriculum.

**Table 1:** Five-Point Likert Scale Categories Used in the Survey

Likert Scale	
Response Categories	Very boring
	Boring
	Neutral (Sometimes boring sometimes interesting)
	Interesting
	Very interesting

The questionnaire also collected information on preferred learning resources, the number of academic sessions conducted per week, the teaching method perceived to impart the maximum knowledge, overall satisfaction with the existing teaching schedule, and suggestions for improving postgraduate teaching through an open-ended feedback section. The finalized questionnaire required approximately five minutes to complete.

Implementation of the survey

The survey was disseminated nationally through multiple academic and professional channels, including Heads of Departments of recognized medical colleges and institutions offering postgraduate anaesthesiology programmes, postgraduate student groups, faculty academic networks, and professional social media platforms. The authors facilitated nationwide dissemination of the survey among eligible participants through these established academic networks. Data collection commenced on November 16, 2023 and continued until the required sample size was achieved.

Outcome measures

The primary outcome of the study was to evaluate postgraduate anaesthesiology students' perceptions of different teaching methods in terms of engagement, effectiveness, and satisfaction, and to characterize their overall teaching preferences. Secondary outcomes included identifying the most and least preferred teaching methods, evaluating students' preferred learning resources and class frequency, assessing geographical variations in perceptions, and analysing open-ended feedback to identify opportunities for improving postgraduate teaching.

Statistical analysis

The sample size for the survey was determined with an alpha error set at 5%, considering a population of eligible participants around 25,000. Using Cochran’s formula for categorical data, the exact sample size was calculated to be 382. To account for incomplete responses, exclusion of ineligible participants, and to improve the precision of the survey findings, the target sample size was increased threefold to 1,146 [15,16].

The dependent variables included students' perceptions of different teaching methods in terms of engagement, effectiveness, and satisfaction, preferred learning resources, preferred class frequency, and feedback on improving postgraduate teaching. The independent variables included the type of teaching method, year of postgraduate training, geographical region, number of postgraduate teaching sessions per week, and preferred mode of learning.

Continuous variables were expressed as mean±standard deviation (SD), while categorical variables were presented as frequencies and percentages. Comparisons between categorical variables were performed using the Chi-square test, whereas continuous variables were analysed using one-way analysis of variance (ANOVA). Multivariable regression analysis was performed to identify factors independently associated with student satisfaction. A two-sided p value <0.05 was considered statistically significant. Statistical analyses were performed using IBM SPSS Statistics for Mac (Version 22.0; IBM Corp., Armonk, NY, USA).

AI implementation

As an exploratory component of the study, ChatGPT-4o (OpenAI, San Francisco, CA, USA) was used to generate an illustrative teaching schedule based on aggregated student preferences. Aggregated inputs included student satisfaction ratings for different teaching methods, preferred number of teaching sessions per week, preferred teaching days, and the calendar month for which the schedule was to be prepared. Based on these inputs, ChatGPT-4o generated a proposed teaching schedule.

## Results

The survey was conducted between November 16, 2023 and August 10, 2024 and received 1,293 responses. Of these, 79 respondents did not meet the eligibility criteria and 40 submitted incomplete questionnaires; therefore, a total of 1,174 responses were included in the final analysis. Participants from postgraduate anaesthesiology programmes and senior residency across India contributed to the survey, with the highest participation from Uttar Pradesh (22.4%), followed by Maharashtra (10.3%) and Rajasthan (9.9%) (Figure 1A). The study cohort comprised postgraduate trainees from all years of training and senior residents, with third-year junior residents constituting the largest group (39.9%) (Figure 1B). Most respondents were pursuing an MD in Anaesthesiology (94.5%) (Figure 1C).

**Figure 1:**
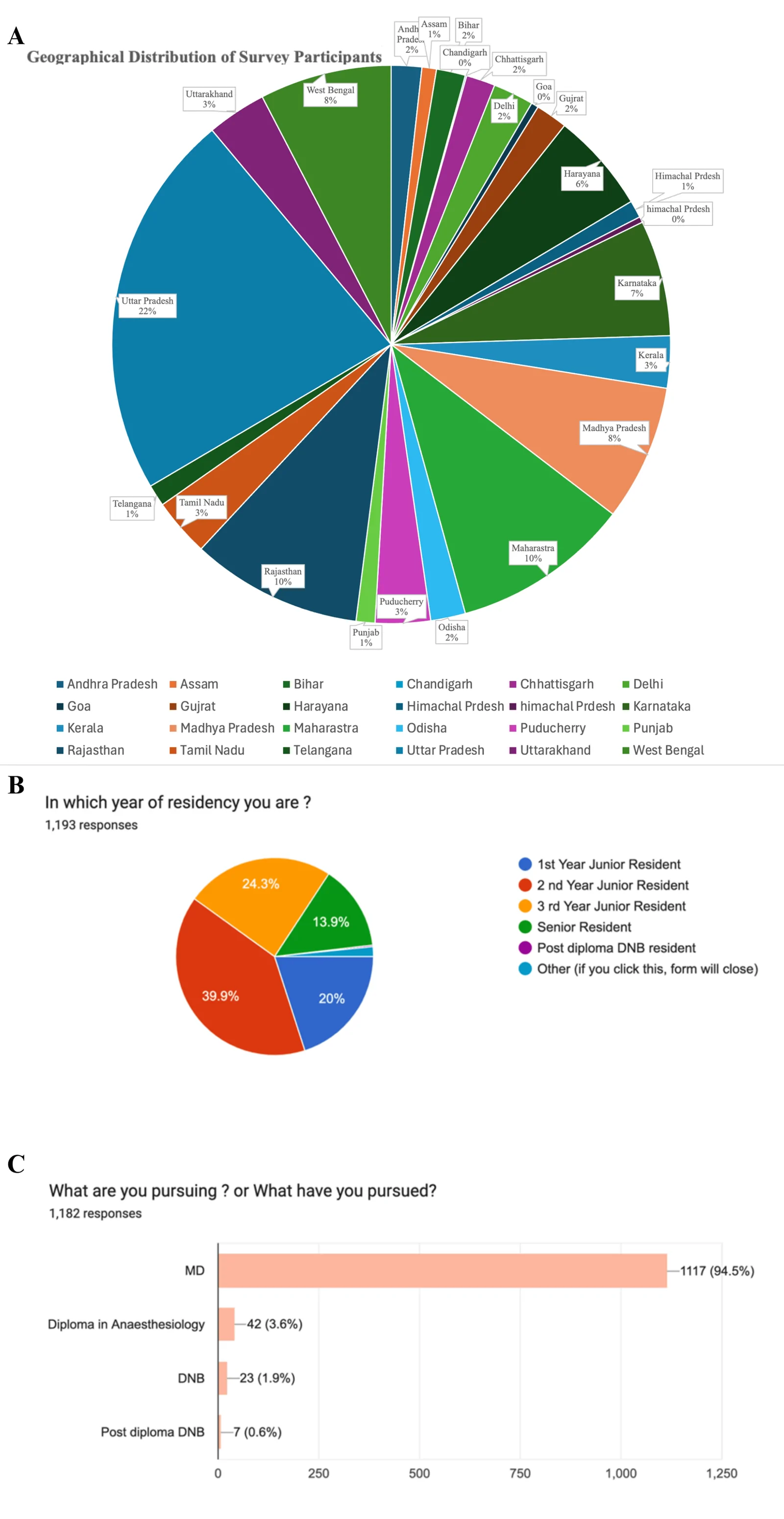
A. Geographical Distribution of Survey Participants. B. Distribution of Participants Based on Year of Junior or Senior Residency. C. Details of Academic Program Enrolment Among Survey Participants.

The primary preferred method of learning was physical textbooks (68.5%), followed by YouTube lectures (31.4%) and e-books (14.3%). However, geographical variations were observed, with e-books being the preferred mode of learning among students in Tamil Nadu (54.2%) and Puducherry (47.6%).

Analysis of class frequency, as reported by 1,148 participants, revealed that the median number of classes per week was 2 (interquartile range (IQR): 1-3). The most commonly reported frequency was one class per week (31.23%). However, significant geographical differences were observed. In southern states (Tamil Nadu, Kerala, Karnataka), students were significantly more likely to attend three or more classes per week compared to their counterparts in central and northern states (Uttar Pradesh, Madhya Pradesh, Rajasthan), where the frequency was predominantly one to two classes per week (odds ratio (OR): 2.14, 95% confidence interval (CI): 1.62-2.83, p<0.001). Student satisfaction regarding class frequency differed significantly according to the number of classes attended per week (χ²(6)=290.66, p<0.001). The distribution of satisfaction across the different class-frequency categories is presented in Table 2. Post-hoc pairwise comparisons using two-proportion z-tests with Bonferroni correction for multiple comparisons showed significant differences among the class-frequency groups, with three classes per week having the highest observed satisfaction (Table 3). Multivariable binary logistic regression further demonstrated that class frequency remained independently associated with satisfaction after adjustment for year of training. Compared with one class per week, the adjusted odds of satisfaction were significantly higher with two classes (adjusted OR (aOR) 4.73, 95% CI 3.39-6.59), three classes (aOR 5.32, 95% CI 3.53-8.02), and more than three classes per week (aOR 7.74, 95% CI 5.39-11.12; all p<0.001).

**Table 2:** Analysis of Student Satisfaction regarding Class Frequency in Anaesthesiology Teaching

Number of classes per week	Frequency	Student Satisfaction regarding the frequency of classes		
		Less than what needed	Satisfied	More than what needed
1	372	201	157	14
2	308	128	162	18
3	169	14	144	11
More than 3	299	16	212	71

**Table 3:** Post-hoc Pairwise Comparison of Student Satisfaction Using Two-Proportion Z-Tests With Bonferroni Correction Pairwise comparisons of the proportion of satisfied students were performed using two-proportion z-tests with Bonferroni correction for six multiple comparisons following a significant overall chi-square test (χ²(6)=290.66, p<0.001). Satisfaction was highest among students attending three classes per week (85.2%) and was significantly higher than in all other class-frequency groups, indicating that three classes per week was associated with the highest student satisfaction regarding class frequency.

Group 1	Group 2	Satisfied in Group 1, %	Satisfied in Group 2, %	Difference in proportions	z-value	Bonferroni-adjusted p-value
1 class/week	2 classes/week	42.2	52.6	−0.104	−2.70	0.041
1 class/week	3 classes/week	42.2	85.2	−0.430	−9.33	<0.001
1 class/week	>3 classes/week	42.2	70.9	−0.287	−7.43	<0.001
2 classes/week	3 classes/week	52.6	85.2	−0.326	−7.10	<0.001
2 classes/week	>3 classes/week	52.6	70.9	−0.183	−4.64	<0.001
3 classes/week	>3 classes/week	85.2	70.9	0.143	3.48	0.003

Considerable variation was observed in the inclusion of different teaching methods within postgraduate teaching rosters. Seminars were the most frequently included teaching method, being part of the teaching roster for 89.4% of respondents, whereas problem-oriented classes were the least frequently included, being reported by only 40.1% of respondents (Figure 2).

**Figure 2:**
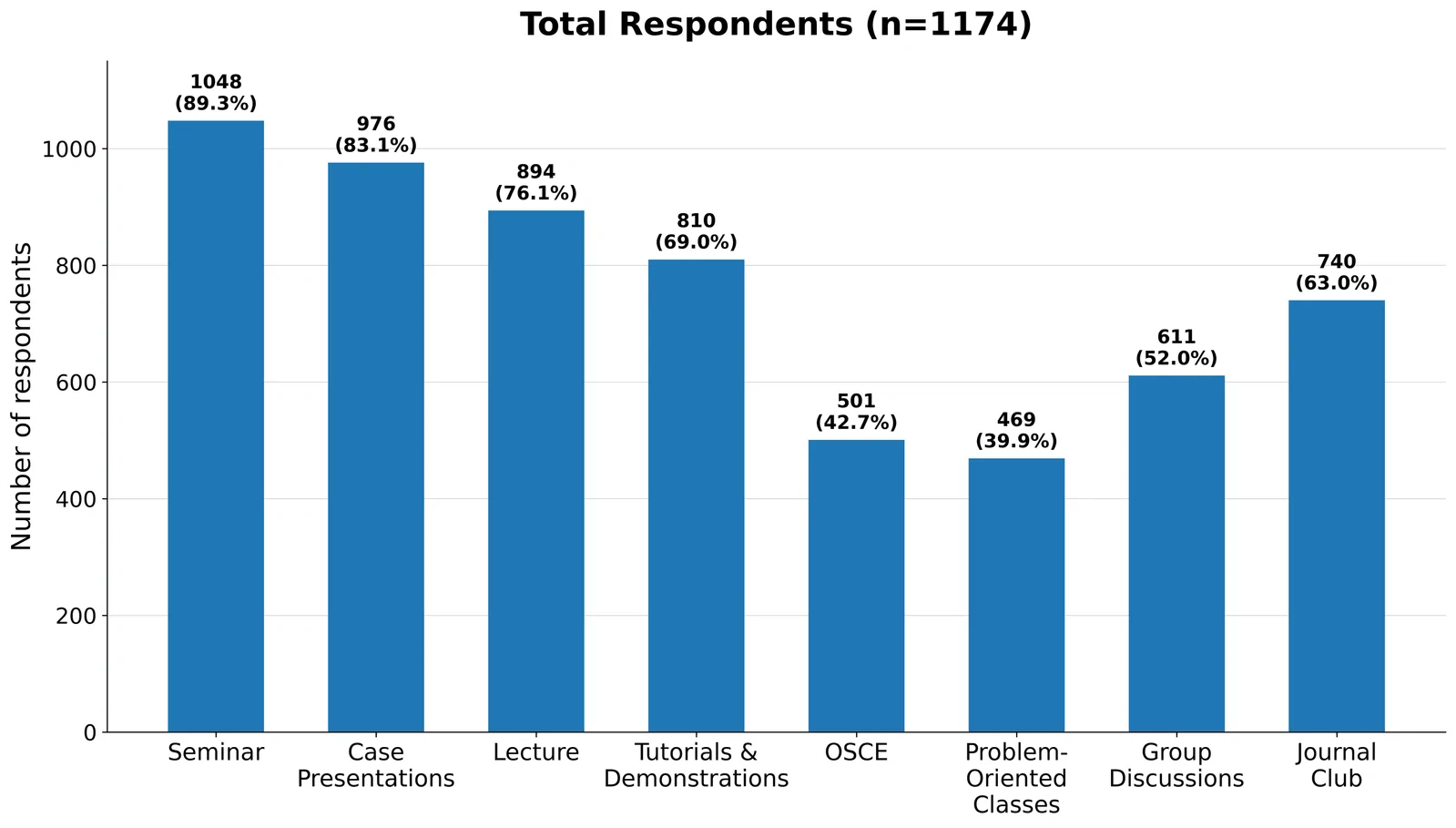
Frequency of Inclusion of Various Teaching Methods in the Postgraduate Teaching Program of Survey Participants

Student perceptions of different teaching methods, assessed using a five-point bidirectional Likert scale, showed that Problem-Oriented Classes received the highest proportion of "Very Interesting" responses (20.5%), whereas Journal Club received the highest proportions of both "Boring" (8.9%) and "Very Boring" (3.6%) responses (Table 4). One-way analysis of variance (ANOVA) demonstrated significant differences in students' perceptions across teaching methods. Post-hoc analysis using Tukey's Honest Significant Difference (HSD) test revealed that Tutorials and Demonstrations had the highest mean Likert score overall and were rated significantly higher than Journal Club, Seminar, Lecture, and OSCE (p<0.05 or p<0.001). No significant difference was observed between Tutorials and Demonstrations and Case Presentations (p=0.99) (Figure 3).

**Table 4:** Analysis of Student Interest and Engagement Across Various Teaching Methods in Anaesthesiology Teaching

Teaching method	Interesting/Very Interesting	Neutral	Boring/Very Boring
Seminar (n=1048)	427 (40.7%)	574 (54.8%)	47 (4.5%)
Lecture (n=894)	485 (54.3%)	375 (41.9%)	34 (3.8%)
Tutorials and Demonstrations (n=810)	615 (75.9%)	184 (22.7%)	11 (1.4%)
Case Presentations (n=976)	705 (72.2%)	246 (25.2%)	25 (2.6%)
Problem-Oriented Classes (n=469)	356 (75.9%)	110 (23.5%)	3 (0.6%)
OSCE (n=501)	357 (71.3%)	140 (27.9%)	4 (0.8%)
Group Discussions (n=611)	437 (71.5%)	168 (27.5%)	6 (1.0%)
Journal Club (n=740)	324 (43.8%)	323 (43.6%)	93 (12.6%)

**Figure 3:**
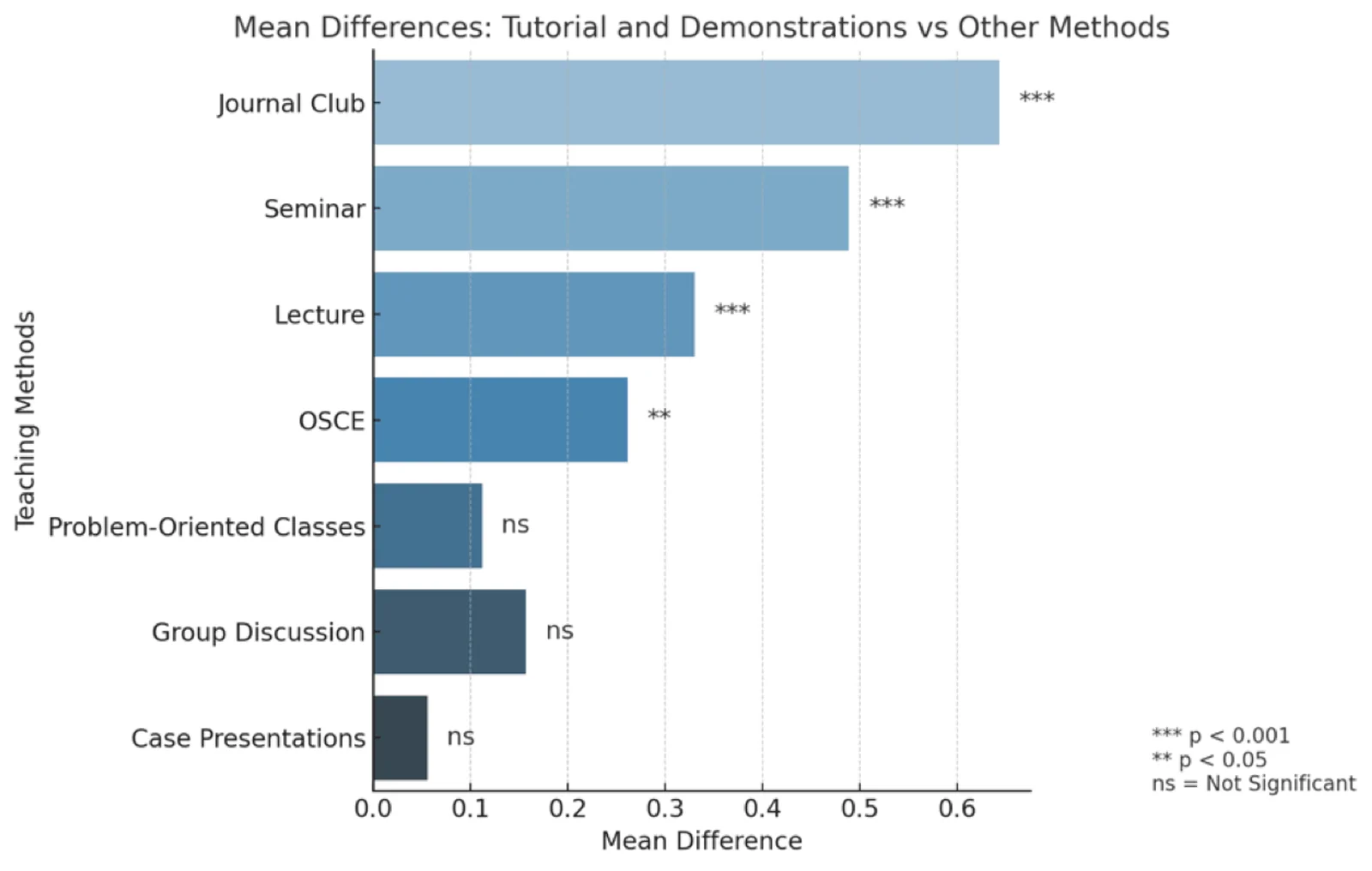
Tukey Honest Significant Difference (HSD) Post Hoc Analysis: Comparison of Tutorial and Demonstrations with Other Teaching Methods

Case Presentations were most frequently identified as the most interesting teaching method, followed by Tutorials and Demonstrations. Case Presentations were also most frequently identified as the teaching method imparting the greatest knowledge, with Tutorials and Demonstrations ranking second. Journal Club and Seminar were the teaching methods most frequently identified as the most boring, with no statistically significant difference between their proportions. This pattern was consistent across all states with more than 40 respondents. Seminar (29.4%) and Journal Club (27%) were also the teaching methods most frequently selected for removal from the teaching schedule to improve postgraduate teaching, with no statistically significant difference between their proportions.

In the survey, it was optional to provide suggestions to make teaching more interesting, and 315 participants offered their input. The most common suggestions included increasing the use of Tutorials and Demonstrations, Case-Based Learning, Group Discussions, Problem-Oriented Classes, and Scenario-Based Learning to promote interactive learning. Participants also suggested incorporating regular mortality meetings, high-risk case discussions, and management planning sessions. Greater emphasis on skill-based training through operation theatre table-side teaching, bedside demonstrations (particularly in the ICU), OSCEs, and simulation-based training was also suggested. Many participants suggested that practical Tutorials and Demonstrations should be conducted by junior faculty, while senior faculty should focus on lectures covering core topics. Additional suggestions included reducing dependence on PowerPoint presentations by incorporating video-based learning, quizzes, and concept maps. Participants from institutes with large postgraduate intakes also suggested dividing students into smaller batches to improve faculty-student interaction.

As an exploratory application of AI, ChatGPT-4o was provided with aggregated student preferences regarding teaching methods and class frequency and was prompted to generate an illustrative two-month postgraduate anaesthesiology teaching schedule. The schedule generated by ChatGPT-4o comprised three teaching sessions per week and assigned greater frequency to the more preferred teaching methods, including Tutorials and Demonstrations, Case Presentations, and Problem-Oriented Classes (Table 5).

**Table 5:** Illustrative AI-Generated Two-Month Teaching Schedule for Anaesthesiology Based on Survey Findings Note: The teaching schedule is designed with a frequency of three classes per week, scheduled on Tuesdays, Thursdays, and Saturdays. OSCE: Objective Structured Clinical Examination.

Week	Tuesday	Thursday	Saturday
Week 1	Tutorial and Demonstration	Problem-Oriented Class	Case Presentation
Week 2	Journal Club	Group Discussion	Case Presentation
Week 3	Tutorial and Demonstration	OSCE	Case Presentation
Week 4	Seminar	Group Discussion	Lecture
Week 5	Tutorial and Demonstration	Problem-Oriented Class	Case Presentation
Week 6	Tutorial and Demonstration	Case Presentation	Case Presentation
Week 7	Tutorial and Demonstration	Tutorial and Demonstration	Case Presentation
Week 8	Seminar	Group Discussion	Lecture

## Discussion

Surveys serve as a key method for gathering self-reported data on beliefs, knowledge, attitudes, opinions, and satisfaction, which are challenging to measure through other approaches. The current national survey investigating postgraduate teaching methods in anaesthesiology provided valuable insights into student perceptions, highlighting the effectiveness of various educational approaches and identifying key areas for improvement. Notably, the highest participation in the current survey was from Uttar Pradesh and Maharashtra, which also have among the highest numbers of postgraduate anaesthesiology seats in India, followed by Rajasthan, another state with substantial postgraduate training capacity [1]. Based on the survey findings, Problem-Oriented Classes, Tutorials and Demonstrations, and Case Presentations were perceived by postgraduate students as the most effective teaching methods for enhancing engagement and knowledge.

The concept of andragogy, which emphasizes that adults and children learn differently, was first introduced by Malcolm Knowles and has since been supported by numerous studies [17-19]. Knowles emphasized principles such as problem-centered learning, relevance to real-life applications, and leveraging prior knowledge, all of which align closely with the teaching methods perceived by postgraduate students as more effective in this survey [17].

In 2019, the Medical Council of India (MCI), later replaced by the National Medical Commission (NMC), introduced Competency-Based Medical Education (CBME) for undergraduate MBBS (Bachelor of Medicine and Bachelor of Surgery) students, emphasizing interactive teaching and active student involvement over traditional didactic methods [20]. However, unlike the undergraduate MBBS curriculum, postgraduate teaching lacks a standardized framework defining the implementation and relative emphasis of lectures, seminars, tutorials, demonstrations, and case presentations. Consequently, teaching practices vary considerably across institutions and are often based on local practices or long-established departmental traditions rather than international postgraduate medical education frameworks [21-23]. Furthermore, to date, no study has systematically compared various teaching methodologies in postgraduate medical education.

The present survey evaluated the teaching methods included in the National Medical Commission postgraduate curriculum and some additional methods being used by institutions, such as Problem-Oriented Classes and OSCE. Although OSCE is primarily an assessment tool, it was included as a teaching method as well in the institutions, and published literature has also described its role in facilitating learning through structured feedback, deliberate practice, and formative assessment within postgraduate teaching programmes [6,7].

The findings of this study align with the principles of adult learning described by Palis and Quiros, who emphasized designing medical education to be relevant, interactive, and connected to learners' prior knowledge and experiences [24]. These principles are further supported by the scoping review of McInerney and Green-Thompson, which highlights the need for innovative and interactive teaching methods in postgraduate medical education and identifies case-based learning (CBL), problem-based learning (PBL), and simulation-based methods as effective approaches for bridging theory and practice [25]. Published literature further supports the superiority of case-based learning over traditional methods in postgraduate education. Bi et al. also demonstrated that students exposed to CBL showed significantly better performance in clinical reasoning and problem-solving than those taught through lectures [26]. These observations support the findings of the present survey and reinforce the integration of interactive, student-centered teaching strategies into postgraduate medical education.

In the current study, AI was used to generate an illustrative two-month teaching schedule based on the survey findings. This exploratory application demonstrates the potential of AI to assist in developing educational schedules based on learner preferences and aligns with published literature describing its role in supporting educational planning and personalized learning [27-29].

This survey, while providing valuable insights into postgraduate teaching methods in anaesthesiology, has certain limitations. First, the survey relied on self-reported data, which may be subject to recall and social desirability biases. In addition, variations in the implementation of teaching methods across institutions and geographical regions may have influenced participants' perceptions rather than reflecting the intrinsic effectiveness of the methods themselves. The study was conducted exclusively among postgraduate students in anaesthesiology, limiting the generalizability of the findings to other medical specialties. Moreover, the survey did not compare the effectiveness of teaching methods based on objective measures of competency gain or learning outcomes. Future research should focus on the objective evaluation of competency development using different teaching methods.

Recommendations

To strengthen postgraduate anaesthesiology teaching in India, the authors recommend actively involving students in the design and periodic review of teaching programmes. Greater emphasis should be placed on interactive teaching methods to promote practical, learner-centred education. Institutions should prioritize small-group, skill-based sessions to facilitate better faculty-student interaction and periodically obtain structured feedback from postgraduate students. AI-based tools may also be explored to assist in developing teaching schedules based on institutional requirements and student feedback.

## Conclusions

Postgraduate anaesthesiology students perceived teaching methods differently, with interactive teaching methods being rated as more interesting than didactic methods. Teaching programmes should incorporate student feedback to enhance learner engagement. Institutions should also conduct regular structured feedback to understand postgraduate students' perceptions of teaching practices at their respective institutions.

## Appendices

Complete survey questionnaire

Cultivating Excellence: An In-Depth Survey on Student Satisfaction with Contemporary Post-Graduate Teaching Methods in Anaesthesiology

Instructions

"Kindly take a moment to fill out this brief 2-minute survey and contribute to the ongoing efforts for improvement." Survey is designed to help improve and elevate the quality of post-graduate teaching methods in the field of anaesthesiology, with a focus on ensuring that students are satisfied and receive high-quality education.

Our commitment! Your name and email address will be kept confidential and will not be shared with any data analysts. But the data provided by you would be utilized for research and analysis. Filling the form would be considered as a positive consent for the same.

1. Name*

________________________________________________________________________________

2. Email id*

________________________________________________________________________________

4. In which State/UT you are pursuing residency PG course?*

○ Andhra Pradesh

○ Arunachal Pradesh

○ Assam

○ Bihar

○ Chhattisgarh

○ Goa

○ Gujarat

○ Haryana

○ Himachal Pradesh

○ Jharkhand

○ Karnataka

○ Kerala

○ Madhya Pradesh

○ Maharashtra

○ Manipur

○ Meghalaya

○ Mizoram

○ Nagaland

○ Odisha

○ Punjab

○ Rajasthan

○ Sikkim

○ Tamil Nadu

○ Telangana

○ Tripura

○ Uttar Pradesh

○ Uttarakhand

○ West Bengal

○ Andaman and Nicobar Islands

○ Chandigarh

○ Dadra and Nagar Haveli and

○ Daman & Diu

○ Delhi NCR

○ Jammu & Kashmir

○ Ladakh

○ Lakshadweep

○ Puducherry

5. In which year of residency you are?*

○ 1st Year Junior Resident

○ 2nd Year Junior Resident

○ 3rd Year Junior Resident

○ Senior Resident

○ Post diploma DNB resident

○ Other (if you click this, form will close)

6. What are you pursuing? or What have you pursued?

☐ MD

☐ Diploma in Anaesthesiology

☐ DNB

☐ Post diploma DNB

Your method of studying anaesthesiology

As the technology is advancing its time to review our teaching methods and their perception to the students. So requesting you to please complete this two minute survery

7. What is your preferred method of learning?*

○ Other:

○ Physical Textbook

○ E-book

○ Journal Articles

○ App

○ Youtube lectures

8. What is your second preferred method of learning?*

○ Other:

○ Physical Textbook

○ E-book

○ Journal Articles

○ App

○ YouTube lectures

9. Ranking of your preferred method of learning? (optional)

Rank as per the preferance (if you prefer YouTube lectures the most give it 1st rank) Note: Don't repeat same rank for two methods otherwise form will not move to next step, means one can give 1st rank only to one method so is rank two and so on...

1

2

3

4

5

6

7

Textbook (physical or ebook)

○

○

○

○

○

○

○

YouTube lectures

○

○

○

○

○

○

○

Online ppt

○

○

○

○

○

○

○

Article and journal

○

○

○

○

○

○

○

Class room notes

○

○

○

○

○

○

○

Departmental classes

○

○

○

○

○

○

○

Other (App/learning programs)

○

○

○

○

○

○

○

10. How many Postgraduate teaching class do you have per week?

○ 1

○ 2

○ 3

○ More than 3

11. Are you satisfied with the number of PG teaching classes conducted?

○ Yes

○ More than what needed

○ Less than what needed

Your perception regarding teaching methodology

As the era is changing with more technology and online methods available, authors want to know how do yo perceive the well known teaching methods in current scenario. So please respond the questions with honesty.

12. Do you have seminars?*

○ Yes

○ No - Skip to question 14

Your perception regarding seminar

13. How do you perceive a seminar?

○ Very boring

○ Boring

○ Neutral (Sometimes boring, sometimes interesting)

○ Interesting

○ Very interesting

Lectures

Lectures considered to be the integral part of teaching methods. Doctors still remember their teachers for fantastic lectures. Simultaneously remember those teachers in too in whom students use to sleep and attend the class for attendance only.

In current scenario, how do you perceive lecture as a teaching method for post graduate teaching, please answer honestly.

14. Do you have lectures?*

○ Yes

○ No - Skip to question 16

Your perception regarding lectures

15. How do you perceive the lecture?

○ Very Boring

○ Boring

○ Neutral (Sometimes boring sometimes interesting)

○ Interesting

○ Very Interesting

Tutorials and Demonstrations

Demonstrations of equipments, procedures is considered to be of much importance. Please mention your perception regarding demonstrations honestly

16. Do you have tutorials and demonstration classes ? *

○ Yes

○ No - Skip to question 18

Your perception regarding demonstrations and tutorials !

17. How do you perceive demonstration and tutorial class?

○ Very boring

○ Boring

○ Neutral (Sometimes boring sometimes interesting)

○ Interesting

○ Very interesting

Case presentations

A difficult case is presented in front of all teachers and colleagues, considered to be of utmost importance. How do you perceive this in current scenario of competency based learning

18. Do you have case presentation classes ? *

○ Yes

○ No - Skip to question 20

Your perception regarding case presentation class

19. How do you perceive case presentation class?

○ Very boring

○ Boring

○ Neutral (Sometimes boring, sometimes interesting)

○ Interesting

○ Very interesting

Problem-oriented class

A hypothetical case with subset of problems is presented for the potential learning. Please submit your response honestly.

20. Do you have problem-oriented classes? *

○ Yes

○ No - Skip to question 22

Your perception regarding problem-oriented classes

21. How do you perceive problem-oriented classes?

○ Very boring

○ Boring

○ Neutral (Sometimes boring, sometimes interesting)

○ Interesting

○ Very Interesting

OSCE (objective structured clinical examination)

You find a pre-structured case history and examination and/or lab reports. So how do you perceive it

22. Do you have OSCE (objective structured clinical examination) in PG teaching program ? *

○ Yes

○ No - Skip to question 24

Your perception regarding OSCE

23. How do you perceive OSCE ?

○ Very boring

○ Boring

○ Neutral (Sometimes boring sometimes interesting)

○ Interesting

○ Very Interesting

Group Discussion

Group leader or facilitator introduced a case scenario based on the principles to be discussed that day and encouraged the group leader to take over the discussion. Please answer honestly about your percet

24. Do you have Group discussions as a part of PG teaching ? *

○ Yes

○ No - Skip to question 26

Your perception regarding Group Discussion

25. How do you perceive Group Discussion ?

○ Very boring

○ Boring

○ Neutral (Sometimes boring sometimes interesting)

○ Interesting

○ Very Interesting

Journal Club

A journal club is an educational meeting in which a group of individuals discuss published articles, providing a forum for a collective effort to keep up with the current literature.

26. Do you have journal clubs as a part of your PG teaching *

○ Yes

○ No - Skip to question 28

Your perception regarding Journal Club

27. How do you perceive journal club ?

○ Very boring

○ boring

○ Neutral (Sometimes boring sometimes interesting)

○ Interesting

○ Very interesting

Summary

28. Which teaching method provide maximum knowledge? *

○ Other:

○ Seminar

○ Demonstration and tutorial

○ Case presentation

○ OSCE

○ Lecture

○ Group Discussion

○ Problem oriented class

○ Journal club

29. Among all teaching method which one is most boring? *

○ Seminar

○ Tutorial and Demonstration

○ Case presentation

○ OSCE

○ Lecture

○ Group Discussion

○ Journal club

○ Problem Oriented class

30. Among all teaching method which one is most interesting ? *

○ Seminar

○ Tutorial and Demonstration

○ Case presentation

○ OSCE

○ Lecture

○ Group Discussion

○ Journal club

○ Problem Oriented class

31. if you are supposed to plan the teaching? Which teaching method/ methods you will remove, so as to make teaching more interesting (you can select more than one ) *

☐ Seminar

☐ Demonstration

☐ Case presentation

☐ OSCE

☐ Lecture

☐ Group Discussion

☐ Problem based class

☐ Journal club

32. Any suggestions to make the teaching more interesting?

________________________________________________________________________________

________________________________________________________________________________

________________________________________________________________________________

________________________________________________________________________________

Conferences (Optional)

Anesthesia conferences facilitate the exchange of knowledge, highlighting the latest advancements in anesthetic techniques, patient care, and research, driving progress in medical practice and innovation. Nevertheless, attendance often remains limited, particularly during sessions. Can you please provide a concise response?

33. Have you attended any Anaesthesiology conference ?

○ Yes

○ No

Let's raise the standard of conferences with your suggestions.

34. What do you like most in an Anaesthesiology conference?

________________________________________________________________________________

________________________________________________________________________________

________________________________________________________________________________

________________________________________________________________________________

35. What do you dislike most in an Anaesthesiology conference ?

________________________________________________________________________________

________________________________________________________________________________

________________________________________________________________________________

________________________________________________________________________________

36. Your suggestions to improve the standard and attendance in the sessions of the conferences!

________________________________________________________________________________

________________________________________________________________________________

________________________________________________________________________________

________________________________________________________________________________
